# Human Papillomavirus–Attributable Cancers — United States, 2012–2016

**DOI:** 10.15585/mmwr.mm6833a3

**Published:** 2019-08-23

**Authors:** Virginia Senkomago, S. Jane Henley, Cheryll C. Thomas, Jacqueline M. Mix, Lauri E. Markowitz, Mona Saraiya

**Affiliations:** ^1^Division of Cancer Prevention and Control, National Center for Chronic Disease Prevention and Health Promotion, CDC; ^2^Division of Viral Diseases, National Center for Immunization and Respiratory Diseases, CDC.

Human papillomavirus (HPV) causes nearly all cervical cancers and some cancers of the vagina, vulva, penis, anus, and oropharynx (*1*).* Most HPV infections are asymptomatic and clear spontaneously within 1 to 2 years; however, persistent infection with oncogenic HPV types can lead to development of precancer or cancer (*2*). In the United States, the 9-valent HPV vaccine (9vHPV) is available to protect against oncogenic HPV types 16, 18, 31, 33, 45, 52, and 58 as well as nononcogenic types 6 and 11 that cause genital warts. CDC analyzed data from the U.S. Cancer Statistics (USCS)^†^ to assess the incidence of HPV-associated cancers and to estimate the annual number of cancers caused by HPV, overall and by state, during 2012–2016 (*3*,*4*). An average of 43,999 HPV-associated cancers were reported annually, and an estimated 34,800 (79%) of those cancers were attributable to HPV. Of these 34,800 cancers, an estimated 32,100 (92%) were attributable to the types targeted by 9vHPV, with 19,000 occurring among females and 13,100 among males. The most common were cervical (9,700) and oropharyngeal cancers (12,600). The number of cancers estimated to be attributable to the types targeted by 9vHPV ranged by state from 40 to 3,270 per year. HPV vaccination is an important strategy that could prevent these cancers, but during 2018, only half of adolescents were up to date on HPV vaccination (*5*). These surveillance data from population-based cancer registries can be used to inform the planning for, and monitor the long-term impact of, HPV vaccination and cancer screening efforts nationally and within states.

CDC analyzed cancer incidence data from USCS, which includes cancer registry data from CDC’s National Program of Cancer Registries and the National Cancer Institute’s Surveillance, Epidemiology, and End Results Program. Data from the District of Columbia (DC) and all states met high-quality data criteria for 2012–2016, covering 100% of the U.S. population. Invasive cancer cases were classified by anatomic site using the *International Classification of Diseases for Oncology, Third Edition* (ICD-O–3)^§^ (Supplementary Table, https://stacks.cdc.gov/view/cdc/80649) and were histologically confirmed. Cancers are not tested for HPV in most cancer registries; therefore, HPV-associated cancers were defined as invasive cancers at anatomic sites with cell types in which HPV DNA frequently is found, including carcinomas of the cervix (i.e., squamous cell cancers [SCC], adenocarcinomas, and other carcinomas) and SCC of the vulva, vagina, penis, oropharynx, and anus (including rectal SCC) (*4*). Oropharyngeal SCC included squamous cell cancer types at the base of tongue, pharyngeal tonsils, anterior and posterior tonsillar pillars, glossotonsillar sulci, anterior surface of soft palate and uvula, and lateral and posterior pharyngeal walls. Anal SCC also included rectal SCCs because they are biologically similar and might be misclassified.^¶^

HPV-associated cancer incidence rates were calculated using reported cases as the numerator and modification of annual county population estimates as the denominator,** standardized to the 2000 U.S. standard population and expressed as cases per 100,000 persons. The USCS data, including the numbers and rates of HPV-associated cancers, are available to the public through the USCS Data Visualizations Tool.^††^ To estimate the number of HPV-attributable cancers (cancers that are probably caused by HPV), the average annual number of HPV-associated cancers was multiplied by the percentage of each cancer type found to be attributable to HPV in a large U.S. study using HPV genotyping (*3*). Estimates of HPV-attributable cancers were rounded to the nearest 100 for national data and to the nearest 10 for state-level data. Cancers were grouped as those attributable to the types targeted by 9vHPV, to other HPV types, and HPV-negative cancers (those that occur at anatomic sites in which HPV-associated cancers are often found but do not have detectable HPV DNA). The percentage of HPV-negative cancers was calculated as the difference between the total HPV-associated cancers and the HPV-attributable estimates.

During 2012–2016, an average of 43,999 HPV-associated cancers (12.2 per 100,000 persons) were reported annually, and an estimated 79% (34,800) of these cancers were attributable to HPV (Table 1). Of these cancers, an estimated 32,100 (92%) were attributable to the types targeted by 9vHPV. The largest number were oropharyngeal cancer (12,600), followed by cervical (9,700), anal (6,000), vulvar (2,500), penile (700), and vaginal cancers (600). Among cancers estimated to be attributable to the types targeted by 9vHPV, 19,000 (59%) occurred among females, and 13,100 (41%) occurred among males.

**TABLE 1:** Average annual number and rate of human papillomavirus (HPV)–associated cancers and estimated percentage and annual number of cancers attributable to HPV, by HPV type, cancer type, and sex — United States,^*^ 2012–2016

Cancer type	Reported HPV-associated cancers^†^		Estimated no.^§^ (%) of cancers attributable to HPV types^¶^		
	Total no.**	Rate^††^	9vHPV-targeted	Other HPV	HPV-negative
**Cervix**	12,015	7.2	9,700 (81)	1,200 (10)	1,100 (9)
**Vagina**	862	0.4	600 (73)	0 (2)	300 (25)
**Vulva**	4,009	2.1	2,500 (63)	300 (6)	1,200 (31)
**Penis**	1,303	0.8	700 (57)	100 (6)	500 (37)
**Anus**	6,810	1.8	6,000 (88)	200 (3)	600 (9)
Female	4,539	2.3	4,100 (90)	100 (2)	300 (8)
Male	2,270	1.3	1,900 (83)	100 (6)	300 (11)
**Oropharynx**	19,000	4.9	12,600 (66)	900 (5)	5,500 (29)
Female	3,460	1.7	2,100 (60)	100 (3)	1,300 (37)
Male	15,540	8.5	10,500 (68)	800 (5)	4,200 (28)
**Total**	**43,999**	**12.2**	**32,100 (73)**	**2,700 (6)**	**9,200 (21)**
**Female**	**24,886**	**13.7**	**19,000 (76)**	**1,700 (7)**	**4,200 (17)**
**Male**	**19,113**	**10.6**	**13,100 (69)**	**1,000 (5)**	**5,000 (26)**

**Abbreviations:** 9vHPV = 9-valent HPV vaccine; ICD-O-3 = *International Classification of Diseases for Oncology, Third Edition.*

* Compiled from population-based cancer registries that participate in the CDC National Program of Cancer Registries, and/or the National Cancer Institute's Surveillance, Epidemiology, and End Results Program and meet the criteria for high data quality for all years during 2012–2016, covering 100% of the U.S. population.

^†^ HPV-associated cancers were defined as invasive cancers at anatomic sites with cell types in which HPV DNA frequently is found. All cancers were histologically confirmed. Cervical cancers (ICD-O-3 site codes C53.0–C53.9) are limited to carcinomas (ICD-O-3 histology codes 8010–8671, 8940–8941). Vaginal (ICD-O-3 site code C52.9), vulvar (ICD-O-3 site codes C51.0–C51.9), penile (ICD-O-3 site codes C60.0–60.9), anal (ICD-O-3 site codes C20.9, C21.0–C21.9) and oropharyngeal (ICD-O-3 site codes C01.9, C02.4, C02.8, C05.1, C05.2, C09.0, C09.1, C09.8, C09.9, C10.0, C10.1, C10.2, C10.3, C10.4, C10.8, C10.9, C14.0, C14.2, and C14.8) cancer sites are limited to squamous cell carcinomas (ICD-O-3 histology codes 8050–8084, 8120–8131).

^§^ HPV-attributable cancers are cancers that are probably caused by HPV (https://academic.oup.com/jnci/article/107/6/djv086/872092). Estimates for attributable fraction were based on studies that used population-based data from cancer tissue studies to estimate the percentage of those cancers probably caused by HPV. The estimated number of cancers attributable to HPV was calculated by multiplying the number of reported HPV-associated cancer cases by the percentage of each cancer type attributable to HPV. The total of HPV-attributable cancers is the sum of cancers attributable to types included in the 9vHPV and cancers attributable to other HPV types (e.g. 32,100 + 2,700 = 34,800). Estimated counts were rounded to the nearest 100 (counts <100 are not displayed) and might not sum to total because of rounding.

^¶^ “9vHPV-targeted” types include oncogenic HPV types 16, 18, 31, 33, 45, 52, and 58. “Other HPV” includes other oncogenic HPV types. “HPV-negative” cancers are those that occur at anatomic sites in which HPV-associated cancers are often found, but HPV DNA was not detected.

** The total reported count is the annual count averaged over the 5-year period and might not sum to total because of rounding.

^††^ Rates are per 100,000 persons; age-adjusted to the 2000 U.S. standard population.

The annual number of cancers estimated to be attributable to the types targeted by 9vHPV ranged by state from 40 (Wyoming) to 3,270 (California) (Table 2). Oropharyngeal cancer was the most common cancer estimated to be attributable to types targeted by 9vHPV in most states, except in Texas, where cervical cancer was most common and in Alaska, DC, New Mexico, and New York, where estimates of oropharyngeal and cervical cancers attributable to the types targeted by 9vHPV were the same.

**TABLE 2:** Estimated annual number of human papillomavirus (HPV)–attributable cancers,* by cancer type,**†** HPV type,**^§^** and state — United States,**^¶^** 2012–2016

State	Estimated no.**								
	All cancers			Oropharynx (male and female)			Cervix		
	9vHPV-targeted	Other HPV	HPV-negative	9vHPV-targeted	Other HPV	HPV-negative	9vHPV-targeted	Other HPV	HPV-negative
Alabama	540	40	160	220	10	100	170	20	20
Alaska	60	<10	20	20	<10	10	20	<10	<10
Arizona	530	40	150	220	10	100	160	20	20
Arkansas	360	30	100	140	<10	60	120	10	10
California	3,270	260	870	1,170	80	510	1,120	130	130
Colorado	460	40	130	190	10	80	130	20	20
Connecticut	370	30	110	150	<10	70	100	10	10
Delaware	110	<10	30	40	<10	20	30	<10	<10
District of Columbia	60	<10	10	20	<10	<10	20	<10	<10
Florida	2,690	210	780	1,170	80	520	730	90	90
Georgia	1,050	80	300	400	30	180	320	40	40
Hawaii	120	<10	30	50	<10	20	40	<10	<10
Idaho	150	10	40	60	<10	30	40	<10	<10
Illinois	1,310	100	380	500	30	220	400	50	50
Indiana	740	60	220	300	20	130	210	30	20
Iowa	330	30	100	120	<10	50	90	10	10
Kansas	280	20	80	110	<10	50	80	10	<10
Kentucky	590	50	180	230	10	100	170	20	20
Louisiana	520	40	150	200	10	90	160	20	20
Maine	170	10	60	70	<10	30	30	<10	<10
Maryland	550	40	160	220	10	90	160	20	20
Massachusetts	660	50	210	290	20	130	150	20	20
Michigan	1,000	80	300	410	30	180	260	30	30
Minnesota	470	40	150	200	10	90	120	10	10
Mississippi	350	30	100	130	<10	60	110	10	10
Missouri	710	60	200	290	20	130	200	20	20
Montana	100	<10	30	40	<10	20	30	<10	<10
Nebraska	170	10	50	60	<10	30	50	<10	<10
Nevada	270	20	70	100	<10	50	90	10	10
New Hampshire	140	10	40	70	<10	30	30	<10	<10
New Jersey	880	70	250	320	20	140	290	30	30
New Mexico	170	10	50	60	<10	30	60	<10	<10
New York	1,980	160	530	660	40	290	660	80	80
North Carolina	1,100	90	330	470	30	210	300	40	30
North Dakota	60	<10	20	30	<10	10	10	<10	<10
Ohio	1,260	100	370	500	30	220	360	40	40
Oklahoma	420	30	120	150	<10	70	130	20	20
Oregon	430	30	120	190	10	80	110	10	10
Pennsylvania	1,410	110	420	550	40	240	400	50	50
Rhode Island	110	<10	30	40	<10	20	30	<10	<10
South Carolina	550	40	170	240	20	100	150	20	20
South Dakota	80	<10	20	30	<10	10	20	<10	<10
Tennessee	780	60	220	310	20	130	230	30	30
Texas	2,310	200	620	830	50	360	890	110	100
Utah	160	10	40	60	<10	30	50	<10	<10
Vermont	60	<10	20	30	<10	10	10	<10	<10
Virginia	760	60	220	310	20	140	210	30	20
Washington	690	50	200	280	20	120	190	20	20
West Virginia	250	20	70	100	<10	40	70	<10	<10
Wisconsin	560	40	170	240	20	100	150	20	20
Wyoming	40	<10	10	20	<10	<10	10	<10	<10

**Abbreviations:** 9vHPV = 9-valent HPV vaccine; ICD-O-3 = *International Classification of Diseases for Oncology, Third Edition.*

* HPV-attributable cancers are cancers that are probably caused by HPV (https://academic.oup.com/jnci/article/107/6/djv086/872092). Estimates for attributable fraction were based on studies that used population-based data from cancer tissue studies to estimate the percentage of those cancers probably caused by HPV.

^†^ HPV-associated cancers were defined as invasive cancers at anatomic sites with cell types in which HPV DNA frequently is found. All cancers were histologically confirmed. Cervical cancers (ICD-O-3 site codes C53.0–C53.9) are limited to carcinomas (ICD-O-3 histology codes 8010–8671, 8940–8941). Vaginal (ICD-O-3 site code C52.9), vulvar (ICD-O-3 site codes C51.0–C51.9), penile (ICD-O-3 site codes C60.0–60.9), anal (ICD-O-3 site codes C20.9, C21.0–C21.9), and oropharyngeal (ICD-O-3 site codes C01.9, C02.4, C02.8, C05.1, C05.2, C09.0, C09.1, C09.8, C09.9, C10.0, C10.1, C10.2, C10.3, C10.4, C10.8, C10.9, C14.0, C14.2, and C14.8) cancer sites are limited to squamous cell carcinomas (ICD-O-3 histology codes 8050–8084, 8120–8131).

^§^ “9vHPV-targeted” includes oncogenic HPV types 16, 18, 31, 33, 45, 52, and 58. “Other HPV” includes other oncogenic HPV types. “HPV-negative” cancers are those that occur at anatomic sites in which HPV-associated cancers are often found, but HPV DNA was not detected.

^¶^ Compiled from population-based cancer registries that participate in the CDC National Program of Cancer Registries, and/or the National Cancer Institute's Surveillance, Epidemiology, and End Results Program and meet the criteria for high data quality for all years 2012–2016, covering 100% of the U.S. population.

** The estimated number of HPV-attributable cancers was calculated by multiplying the number of HPV-associated cancer cases by the percentage of each cancer type attributable to HPV. The total of HPV attributable cancers was the sum of cancers attributable to types targeted by 9vHPV and other HPV types. HPV-negative counts were the difference of the total count and the HPV-attributable counts. Estimates were rounded to the nearest 10; counts <10 are not displayed.

## Discussion

Each year during 2012–2016, an estimated average of 34,800 HPV-attributable cancers were diagnosed in the United States, and 92% (32,100) were attributable to the HPV types targeted by 9vHPV. Previous annual estimates of cancers attributable to the types targeted by 9vHPV were 28,500 for 2008–2012 (*4*), 30,000 for 2010–2014,^§§^ and 31,200 for 2011–2015.^¶¶^ The higher estimates in more recent years are, in part, due to an aging and growing population and increases in oropharyngeal, anal, and vulvar cancers (*6*).

HPV vaccination is an important component of cancer prevention, yet only about half of adolescents are up to date on this vaccine (*5*). The Advisory Committee on Immunization Practices recommends routine HPV vaccination at age 11–12 years and catch-up HPV vaccination for all persons through age 26 years. Catch-up vaccination is not recommended for all adults aged >26 years because the benefit of HPV vaccination decreases in older age groups; however, vaccination based on shared clinical decision-making can be considered for some persons aged 27–45 years who are not adequately vaccinated (*7*). In 2018, HPV vaccination coverage varied by state, and no state met the Healthy People 2020 objective for HPV vaccination (receipt of 2 or 3 doses of HPV vaccine by 80% of adolescents aged 13–15 years).*** State efforts to meet the Healthy People 2020 objective for HPV vaccination could reduce geographic disparities in HPV-associated cancer incidence in the future.

Cervical cancer is the only HPV-associated cancer for which screening is routinely recommended. Recommendations state that women aged 21–65 years be screened regularly for cervical precancers and cancers. Women aged 21–29 years should be screened with the Papanicolaou (Pap) test every 3 years. Women aged 30–65 years can be screened with one of three strategies: the Pap test every 3 years, an HPV test every 5 years, or both a Pap and HPV test every 5 years. Regardless of screening strategy, all abnormal test results require follow-up of abnormal results and appropriate treatment (*8*). The Healthy People 2020 target for cervical cancer screening coverage is 93%; however, in 2015 only 81% of women aged 21–65 years reported receiving a Pap test within the past 3 years; coverage was lower among Asians, Hispanics, non–U.S. born, and uninsured women.^†††^

Progression from persistent HPV infection to precancers and eventually invasive cancer occurs over many years, so it might be too soon to see the effects of HPV vaccination on invasive cancers (*2*). However, several studies have demonstrated the population-level impact of HPV vaccination in the United States, including a reduction in the prevalence of vaccine-type HPV infection (*9*) and rates of high-grade cervical precancers in women aged <25 years (*10*). Cervical cancer rates declined 1.6% per year during 1999–2015, largely because of screening, although decreases among the youngest age group of women might be due in part to HPV vaccination (*6*).

The findings in this report are subject to at least one limitation. Although population-based cancer registries provide a reliable system for counting invasive cancers, they do not routinely collect or report information on HPV genotype status in cancer tissue; actual counts of HPV-associated cancers can be provided, but for HPV-attributable cancers, only estimates are available. An important strength of this study, however, is the use of high-quality, population-based surveillance data with 100% coverage of the U.S. population, allowing for specific histologic definitions to monitor HPV-associated cancer incidence nationally and in each state.

Among the 43,999 HPV-associated cancers that occur each year in the United States, an estimated 34,800 are attributable to HPV, including 32,100 attributable to HPV types targeted by 9vHPV. During 2018, only half of adolescents were up to date on HPV vaccination (*5*). Surveillance for HPV-associated cancers using population-based cancer registries with high-quality data and the assessment of HPV-attributable cancers can be used to monitor the long-term impact of HPV vaccination and current cervical cancer screening strategies in the United States. The examination of state-level data enables states to plan for and monitor the impact of vaccination and cervical cancer screening.

**Summary**

**What is already known about this topic?**

Human papillomavirus (HPV) causes nearly all cervical cancers and some cancers of the vagina, vulva, penis, anus, and oropharynx. Cervical cancer screening and HPV vaccination can prevent many of these cancers.

**What is added by this report?**

An average of 34,800 cancers reported annually in the United States during 2012–2016 were attributable to HPV. Of these, 32,100 (92%) cancers were attributable to HPV types targeted by the 9-valent HPV vaccine, ranging by state from 40 to 3,270.

**What are the implications for public health practice?**

Ongoing surveillance for HPV-associated cancers can inform state-level and national-level HPV vaccination and cervical cancer screening efforts and monitor their long-term impact.

## Footnotes

* https://publications.iarc.fr/108.

† https://www.cdc.gov/cancer/uscs.

§ http://www.iacr.com.fr/index.php?option=com_content&view=category&layout=blog&id=100&Itemid=577.

¶ https://journals.lww.com/jlgtd/Fulltext/2019/04001/2019_ASCCP_Oral_Presentation_Abstracts.2.aspx.

** https://seer.cancer.gov/popdata.

†† https://www.cdc.gov/cancer/dataviz, June 2019.

§§ https://www.cdc.gov/cancer/hpv/pdf/USCS-DataBrief-No1-December2017-508.pdf.

¶¶ https://www.cdc.gov/cancer/hpv/pdf/USCS-DataBrief-No4-August2018-508.pdf.

*** https://www.cdc.gov/vaccines/imz-managers/coverage/teenvaxview/data-reports/hp2020/index.html.

††† https://www.healthypeople.gov/2020/topics-objectives/.
